# Animal welfare definitions, frameworks, and assessment tools: Advancing the measurement and laying the foundation for improved animal welfare through a three-step approach

**DOI:** 10.1017/awf.2025.23

**Published:** 2025-05-07

**Authors:** F Josef van der Staay, Vivian C Goerlich, Franck LB Meijboom, Saskia S Arndt

**Affiliations:** 1 Department of Population Health Sciences, Division of Farm Animal Health, Behaviour and Welfare Group (Formerly: Emotion and Cognition Group), Faculty of Veterinary Medicine, University Utrecht, Utrecht, The Netherlands; 2 Department of Population Health Sciences, Division of Animals in Science and Society, Animal Behaviour Group, Faculty of Veterinary Medicine, Utrecht University, Utrecht, The Netherlands; 3 University Medical Center (UMC), Utrecht, Brain Centre, Utrecht, The Netherlands; 4 Department Population Health Sciences, Division of Animals in Science & Society, Sustainable Animal Stewardship, Faculty of Veterinary Medicine, University Utrecht, Utrecht, The Netherlands

**Keywords:** animal welfare, ethics frameworks, Five Domains model, Five Freedoms, Quality of Life, stakeholders

## Abstract

To qualify and quantify animal welfare, novel assessment tools have been and are being developed, while existing assessment tools are being modified so that they can be applied to multiple species living under different housing and management conditions. The results of such assessments should be reliable, consistent and reproducible. We review the steps that should ideally be taken to develop, validate and apply animal welfare assessment tools. The first step should be to find a definition of animal welfare that the various stakeholders can agree upon. The second step should be to formulate and agree upon a framework for the evaluation of animal welfare. Both theoretical/conceptual frameworks, which provide a structure for research and suggest which facets are considered important, and ethical frameworks, which explicate the underlying moral position, should be considered. Finally, animal welfare assessment tools should be developed and validated based on both the adopted welfare definition and the welfare evaluation framework(s). However, this three-step approach has not always been followed in the development of welfare assessment tools currently in use. We expect that transparency and clarity regarding the underlying definitions and frameworks will increase the likelihood that the resulting welfare assessment tools will give similar weight to the aspects considered relevant to animal welfare, as it helps to specify the aspects that are considered to be key elements of animal welfare. This approach should lead to convergent assessment results and higher correlation of welfare indicators between assessment tools.

## Introduction

**Figure 1.**
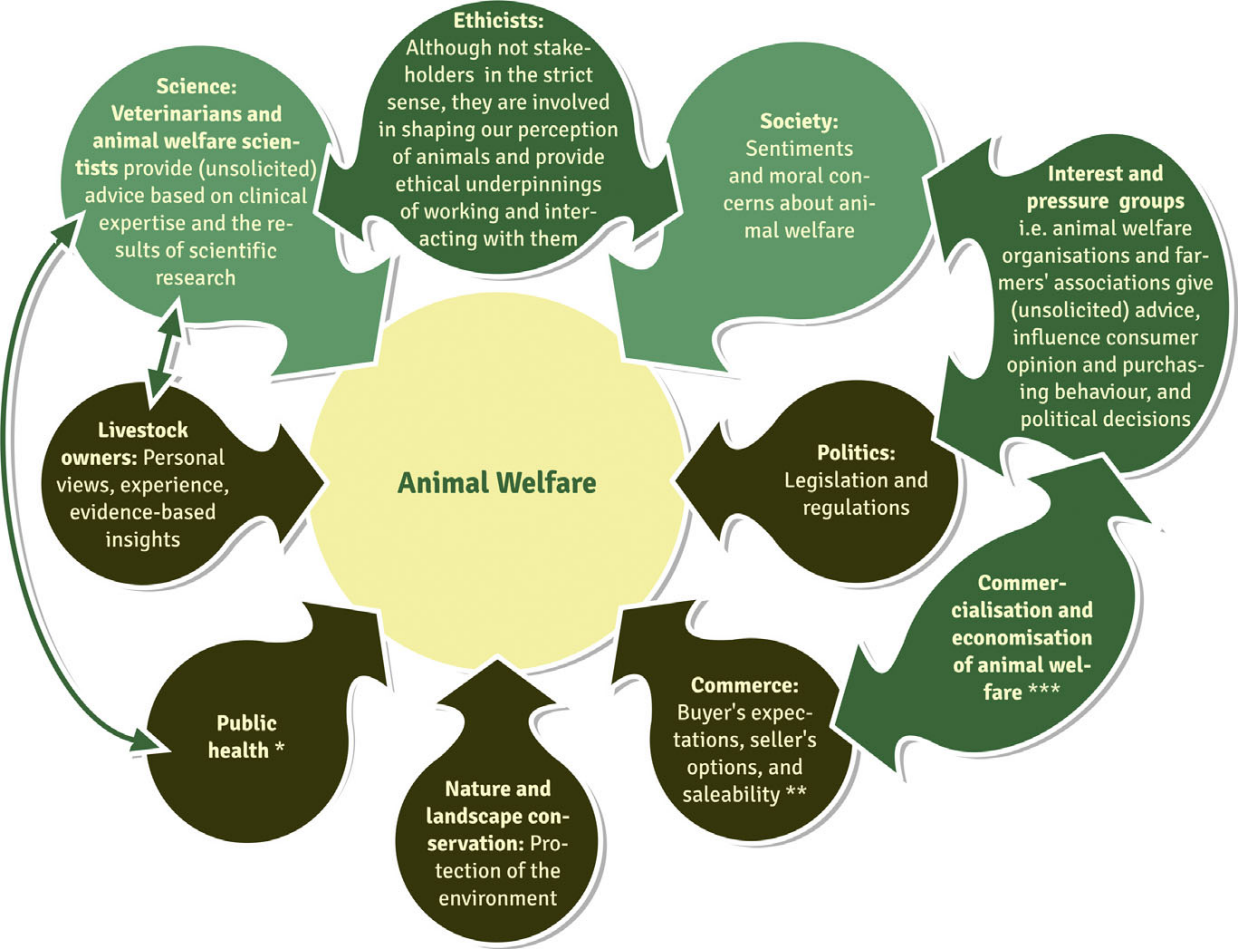
Key stakeholders involved in discussing welfare. *Compromised animal welfare due to restriction or prohibition of outdoor access in order to: (i) reduce air and water pollution; (ii) reduce or prevent airborne or waterborne transmission of (zoonotic) diseases, e.g. by keeping poultry indoors during an outbreak of avian influenza; (iii) hunt, trap or poison for pest control. **With respect to (a) the quality of animal-derived products, e.g. eggs, meat, leather, (b) appearance and characteristics of the animal itself, e.g. compliance with breed standards, (c) animal welfare, e.g. keeping animals in a species-appropriate environment. ***For example through the certification of animal welfare labels by animal welfare organisations and the marketing of these labelled products by retailers. ↔ Strong mutual influences and contacts. (Figure modified and extended from Nordquist *et al.* [2017], originally distributed under a CC BY licence).

Animal welfare is a multifaceted concept consisting of (scientific) knowledge, moral concepts of scientists and society at large (Bayvel & Cross 2010; Miele *et al.* 2011; Nordquist *et al.* 2017), and the activities, actions, and interactions between stakeholders that should culminate in a shared understanding of how to treat animals and ensure their welfare (for an overview of key animal welfare stakeholders, see Figure 1). Concerns about the welfare of animals, such as those kept on intensive farms or used as laboratory animals in biomedical research, are stimulating debate at all levels of society (Ohl & van der Staay 2012; European Commission 2023).

Animal welfare scientists are dedicated to conducting scientific research on the health and welfare of animals, taking action when animal welfare is compromised, providing direction for future developments, and providing policy-makers with science-based information to formulate and implement animal welfare laws and regulations. A major focus of the work of animal welfare scientists is to make animal welfare measurable with the goal of improving it (Fraser 2003), particularly in commercial animal agriculture and laboratory animal research, but ultimately in all contexts where humans can be held accountable.

The European Commission has launched major research initiatives that include animal welfare, such as the Welfare Quality® project (start: 2004, end: 2009), Animal Welfare Indicators (start: 2011, end: 2015) and, most recently, the European Partnership on Animal Health and Welfare (start: 2024). Note that this is not an exhaustive list.

Ideally, three sequential steps are required to assess animal welfare and derive welfare indicators for individual animals and/or groups of animals: the formulation and acceptance of a definition or concept of animal welfare; the choice of a conceptual and possibly ethical framework for animal welfare; and finally the development, validation and application of an animal welfare assessment tool. In practice, however, these steps are not always followed consistently and, in particular, the underlying definition or concept of animal welfare and ethical considerations are often not explicitly addressed and published.

To aid the dialogue and progress on animal welfare we call for unambiguous terminology. Describing the basic characteristics and scope of animal welfare should help to clarify the meaning of the term, so that all subsequent discussions and actions are based upon the same understanding of the term. This is particularly necessary when different stakeholders are trying to agree on how to proceed in order to improve and safeguard animal welfare, i.e. to develop a framework that is detailed enough to measure animal welfare and to derive action guidelines that can be applied in practice. Therefore, we provide an overview on animal welfare concepts, frameworks, and assessment tools, which we critically discuss to clarify aims, applicability and limitations of these different aspects. We also highlight the importance of following the three steps outlined above when developing welfare assessment tools.

Due to the complex nature of the concept of animal welfare, a wide range of definitions have been proposed at a theoretical level (Hewson 2003; Carenzi & Verga 2009; Fisher 2009; Broom 2011). Based, at the very least, on an implicit understanding of animal welfare but, ideally, on a widely accepted definition of animal welfare, animal scientists develop tools to assess animal welfare. Frameworks for developing assessment tools (hereafter called ‘Animal Welfare Evaluation Framework’ [AWEF]) and to take actions for improving animal welfare are, for example, provided by the Five Freedoms concept (Brambell *et al.* 1965) and the Five Domains model (Mellor & Reid 1994). These frameworks have strongly influenced contemporary animal welfare studies and focused attention on factors considered relevant for measuring and improving animal welfare (Blokhuis *et al.* 2010; Hampton *et al.* 2023), albeit with varying degrees of specificity. These and other frameworks provide the conceptual framework and theoretical information for formulating animal welfare guidelines. The aim of these guidelines is to provide a concrete set of instructions on how animals should be treated in order to ensure and improve their welfare.

“*Guidelines describe the best practice agreed at a particular time following consideration of scientific information and accumulated experience. It also reflects society’s values and expectations regarding the care of animals. A guideline is usually a higher standard of care than minimum standards, except where the standard is best practice*” (Burton 2009; p 2). The science-based information ultimately serves the development of policies and strategies to improve animal welfare, once translated into practical measurement tools and guides for action (Webster 2016). The formulation of animal welfare policies and their implementation in practice can be seen as the translation of animal welfare concepts into action. Politicians and policy-makers are sensitive to the opinions and feelings of interest groups and society at large (see Figure 1). They can influence the conditions under which animals are kept by enacting or amending laws and regulations. Practitioners and (commercial) animal owners may recognise that improved animal welfare adds value to many animal-derived products (e.g. Buller & Roe 2014) and services. The impact of policies that are intended to affect welfare is measured using animal welfare assessment tools.

Two decades ago, Fraser (2003) suggested that as a first step in the development of animal welfare assessment tools, it could/should be made more explicit which value assumptions and factors are considered to be of particular importance. We argue that there is little point in developing welfare assessment tools if the underlying definition or concept of animal welfare is not disclosed, as it must be clear what is meant by animal welfare before attempting to measure it (Rault *et al.* 2020). A definition of animal welfare is particularly important because it influences the direction in which welfare assessment tools are developed. We expect that a consensus on definitions and frameworks will make it less likely that the resulting assessment tools will give different weight to different aspects of welfare and show poor agreement on results.

## Animal welfare concepts or definitions (AWC/Ds)

Concepts and frameworks can be interpreted in different ways because, despite nearly 60 years of research on animal welfare, there is still no commonly accepted understanding of what animal welfare means (Czycholl *et al.* 2015). A concept is an abstract idea or principle, a general idea or understanding of something, i.e. it is a mental abstraction that serves as a basic building block underlying principles or sets of thoughts and beliefs. A definition is an attempt to state clearly and precisely the proper, general or particular characteristics of an idea or principle, i.e. it attempts to explain the meaning of a word, phrase or concept. The transition from a definition to a concept is a fluid one.

“*Making genuine improvements in animal welfare requires a definition of ‘welfare’ that everyone can buy into, not a split between a scientific view of welfare and a lay view of welfare*” (Dawkins 2008; p 942). Animal welfare has been conceptualised and defined in a variety of ways. Definitions range from short, one-sentence formulations that are limited to one or a few aspects of the main component(s) thought to describe animal welfare, to complex formulations that include a wider range of aspects thought to characterise welfare. Examples of short definitions include those of Dawkins (2008) and Reimert *et al.* (2023). According to Dawkins, animals have good welfare if they are healthy and “*have what they want*” (2008; p 943). Reimert *et al.* define animal welfare “*as the balance of positive and negative affective states over the period of time of interest, which can span from hours to years to lifelong*” (2023; p 4).

Recently, Arndt and co-authors (2022) proposed the Dynamic Animal Welfare Concept (DAWCon) of ‘positive animal welfare’, an extended version of an earlier concept by Ohl and van der Staay (2012):

*“An individual is likely to be in a positive welfare state if it is mentally and physically capable and has the ability and opportunity to respond appropriately to sporadic or sustained appetitive and adverse internal and external stimuli, events and conditions. Appropriate responses are elements of an animal’s normal behaviour. They enable the animal to cope with and adapt to the demands of the [prevailing] environmental circumstances and to reach a state that it perceives as positive, i.e. that evokes positive emotions”* (slightly modified from Arndt *et al.* 2022).

An abridged version defines animal welfare as follows:

*An animal is likely to experience good [positive] welfare when its social and physical environment allows it to adapt to reach a state that it perceives as positive, without being pushed to or beyond the limits of its adaptability.*

The short definition omits some aspects of the Dynamic Animal Welfare Concept and focuses on two aspects of animal welfare: adaptability and positive emotions. It is clear that animal welfare evaluation frameworks based on either the short or the extended version will differ in their complexity and in the number of aspects considered relevant. A further extension of existing long definitions might even add more aspects that are considered relevant (e.g. the definition of Rault *et al.* [2025], which overlaps in many aspects with the definition of ‘positive welfare’ of Arndt *et al.* [2022]). The coexistence of different definitions and concepts requires scholars to engage intensively, critically, and constructively with the alternatives and to discuss them.

## Animal welfare evaluation frameworks (AWEFs)

“*The choice of a theoretical framework influences where we will be able to go. It is important to get the theory right for reasons of truth and understanding. And it is also important to get a strategy that starts us in the right direction, rather than pointing us down a blind alley*” (Nussbaum 2018; p 5). There are many different definitions of ‘framework’. *The Collins Online Dictionary* defines a framework as “*a particular set of rules, ideas, or beliefs which you use in order to deal with problems or to decide what to do*” (‘Framework’ 2024a), while the *Oxford Learners Dictionary* characterises framework as “*a set of beliefs, ideas or rules that is used as the basis for making judgements, decisions, etc*” (‘Framework’ 2024b). Imenda distinguishes between theoretical and conceptual frameworks. While “*a theoretical framework is the application of a theory, or a set of concepts drawn from one and the same theory, to offer an explanation of an event, or shed some light on a particular phenomenon or research problem*” (Imenda 2014; p 189), “*a conceptual framework may be defined as an end result of bringing together a number of related concepts to explain or predict a given event, or give a broader understanding of the phenomenon of interest – or simply, of a research problem. The process of arriving at a conceptual framework is akin to an inductive process whereby small individual pieces [in this case, concepts] are joined together to tell a bigger map of possible relationships.*” (Imenda 2014; p 189). All current AWEFs can be surmised under conceptual frameworks (see next paragraph for examples).

The formulation of a framework can and is used for different purposes: it can provide a structure for research and suggest which facets are considered important; it can be used to develop evaluation tools to measure the impact of interventions that take these facets into account; and it can be used to compare different questions based on the same facets (Rault *et al.* 2020). According to McGinnis and Ostrom, “*A framework provides the basic vocabulary of concepts and terms that may be used to construct the kinds of causal explanations expected of a theory. Frameworks organise diagnostic, descriptive, and prescriptive inquiry*” (2014; p 1). Imenda states that “*the conceptual or theoretical framework is the soul of every research project. It determines how a given researcher formulates his/her research problem – and how s/he goes about investigating the problem, and what meaning s/he attaches to the data accruing from such an investigation*” (2014; p 185). Unfortunately, frameworks are often characterised by vague sets of concepts with loosely defined or even unidentified relationships, leaving it unclear and unexplained why some concepts and relationships are included while others are excluded (Partelow 2023). Therefore, to aid dialogue within and between groups of stakeholders, clear animal welfare frameworks need to be described and agreed upon.

## Theoretical/conceptual frameworks

Examples of commonly used frameworks, namely the Five Freedoms, the Five Domains model, Quality of Life, and the 3Rs (replacement, reduction and refinement of animal experiments) (for more examples, see Jones *et al.* 2022; Colditz 2023) are discussed below. While the issues addressed in these frameworks are more or less broad, they all share a similar goal of minimising or eliminating pain and distress, with parallel developments for farm and laboratory animals. The pros and cons of the most commonly used AWEFs have been critically discussed (e.g. Five Freedoms, McCulloch 2013; Five Domains model, Hampton *et al.* 2023; Quality of Life, Webster 2016).

### The Five Freedoms, the Five Domains model and the Harms model

More than half a century ago, the Five Freedoms were conceived, focusing on the welfare of animals used in agriculture (Brambell *et al.* 1965). This concept was further developed to serve as a “*framework for the analysis of animal welfare*” (Farm Animal Welfare Council [FAWC] 1979). The Five Freedoms can serve as a basis for an assessment tool to monitor outcome parameters, but do not take into account the longitudinal and dynamic nature of well-being. Furthermore, the expression of normal behaviour is not defined and can be interpreted differently (Browning 2019). Indeed, some behaviours may be undesirable because they may be harmful to the animal or to humans (see also Arndt *et al.* 2022 on the discussion of normal vs natural behaviour). Also, the importance of negative emotions is not adequately considered. Animals are not in a positive welfare state if they do not experience negative emotions. These negative emotions and negative states have an important biological function in triggering the animal to cope with the challenges of its environment and to take actions to restore a positive state (Ohl & van der Staay 2012). It is important that animals can experience negative emotions and negative states as long as they have the ability and opportunity to deal with them appropriately (Arndt *et al.* 2022).

The Five Domains model can be seen as a further development of the Five Freedoms (Voogt *et al.* 2023) and has evolved across the past decade (Mellor & Reid 1994; Mellor & Beausoleil 2015; Mellor 2016; Mellor *et al.* 2020). The Five Domains model includes an animal’s mental state and, most importantly, positive affect. Fraser (2003) contributed by identifying three basic conceptual themes that have since been used in protocols to assess animal welfare, namely the biological functioning of the animal, its affective state and its opportunity to live a natural life. These are themes that have appeared in almost all AWEFs released since the first publication of the Five Freedoms (Brambell *et al.* 1965).

The role that unintentional and indirect harm plays in animal welfare is underemphasised in most current frameworks (Hampton *et al.* 2023). About 1½ decades ago, Fraser and MacRae (2011) published their ‘Harm model’, which draws attention to the impact on animal welfare of either: (1) harming animals as a consequence of keeping them; (2) causing harm intentionally; (3) unintentionally as collateral damage of human activities; (4) indirectly by disrupting ecological systems and natural processes. The authors developed a framework for incorporating these unintended and indirect negative effects of human activities into animal welfare science and ethics. The ‘Harm model’ aids recognising and identifying ethically challenging situations and take action to address these (Fawcett *et al.* 2018).

### Quality of Life

“Quality of Life” (Green & Mellor 2011) or “Life worth living” (Webster 2016) suggests that animal welfare “*involves more than the absence of suffering; it concerns the quality of an animal’s whole relationship with its environment, the way it lives its life*“ (Wemelsfelder 2007; p 1), or, as Broom states, Quality of Life “*usually refers to a characteristic of an individual over a time-scale longer than a few days. It is a state of the individual that will vary from good to bad*” (Broom 2007; p 47).While Quality of Life builds on the importance of positive experiences, the concern remains how to balance positive and negative states as they may have different short- and long-term effects.

### Excursion to animal experimental studies: The 3Rs and their further development

In animal research, the 3Rs aim to minimise pain, fear and distress in experimental animals. This goal can be achieved through reduction, using fewer animals; refinement, reducing or eliminating distress; and replacement, using unconscious animals instead of conscious animals (Russell & Burch 1959; Tannenbaum & Bennett 2015; Tannenbaum 2017). The fundamental aim of the 3Rs is to conduct animal experimentation humanely by reducing or eliminating distress and avoiding pain and should not be seen as a call to avoid animal experimentation. Instead, Russell and Burch argue that the application of the 3Rs should in no way compromise the goals of conducting sound scientific research aimed at furthering science and medicine (Tannenbaum & Bennett 2015), i.e. the 3Rs do not address the ethical justification of animal research.

Since the publication of the 3Rs, new and broader ethical frameworks for the use of animals in research have been proposed, driven by the recognition that the 3Rs principles did not address some ethical issues in sufficient depth, such as the moral justification and responsibility for animal experimentation (see information on the development of the 3Rs in the Supplementary material).

## Ethical frameworks

What our obligations are to protect and improve animal welfare, i.e. how to treat animals properly, must be considered from an ethical point of view, which forms the basis of moral obligations in the treatment of animals (Fraser *et al.* 1997; Fraser & MacRae 2011; Schmidt 2011; Uldahl *et al.* 2022). We need to explicitly determine our moral position, or at least be aware of our implicit moral position, and the consequences this has for our moral obligations regarding the ethical moral status of animals and our moral obligations towards them (Brown 2014).

The development of animal welfare concepts and frameworks is strongly influenced by the prevailing ethical considerations. Ethical theories provide a basis for distinguishing and deciding what actions should be considered right or wrong, while a system of rules, guidelines or principles by which one or more ethical theories are applied constitutes an ethical framework (Fawcett *et al.* 2018). Explicitly stating which ethical theory underpins one’s animal welfare position can influence the direction of the welfare debate and the direction in which welfare science progresses, and the way science is translated into policies. As moral agents, humans are able to ascribe moral status to animals. This moral status should be the basis for considering animals as morally considerable for their own sake and implies that animals have interests that can be harmed and promoted. This moral starting point underlies duties to account for the consequences of human actions for animals and to ensure their welfare and/or integrity (see also Arndt *et al.* 2024). Our interactions and relationships with animals and their consequences for animal welfare call for an ethical debate about our responsibility towards animals (Ohl & van der Staay 2012; Montanari *et al.* 2024).

There are fundamental and controversial differences within and between societies and cultures on these basic moral assumptions regarding the moral status of animals. As a result, moral issues, such as the plurality of views on the moral relevance of animal welfare and human responsibility for animals (‘duty of care’), need to be taken into account in the dialogue on animal welfare. For example, societies may differ as regards which animal species are considered suitable for consumption and/or may assign different statuses to different animal species. In addition to ethical arguments, other types of normative arguments play an important role (e.g. religious values making certain groups of animals untouchable, impure or, at the other extreme, sacred) (Szűcs *et al.* 2012). These arguments can be problematic if we assume that the moral status of animals is context-independent. From this perspective, welfare should ultimately be independent of the value that people/human cultures place on particular species (e.g. farm animals, pets, laboratory animals, companion animals or animals considered pests), as the needs of individual animals will be similar in all contexts, and these are the guiding criterion for animal welfare.

Ethical theories that deal with contentious moral issues seek to achieve at least one of two different goals: (i) to help determine what is morally right or wrong; and (ii) to help identify moral problems and to guide and structure moral debates about them. Apart from different assumptions about what is morally right or wrong, a second source of disagreement between ethical theories concerns differences in understanding of the factors considered relevant to animal welfare, probably due to a gap in (empirical) knowledge about the animal and its environment, such as management and housing conditions, and its ecosystem (Fraser 1999; Davies *et al.* 2020). The effects of the identified factors vary in their importance for animal welfare and should be weighted accordingly. This knowledge informs both ethicists and animal welfare scientists and provides a sound basis for judging which conditions and actions affect animal welfare. It can also help to better substantiate and strengthen moral claims.

**Figure 2.**
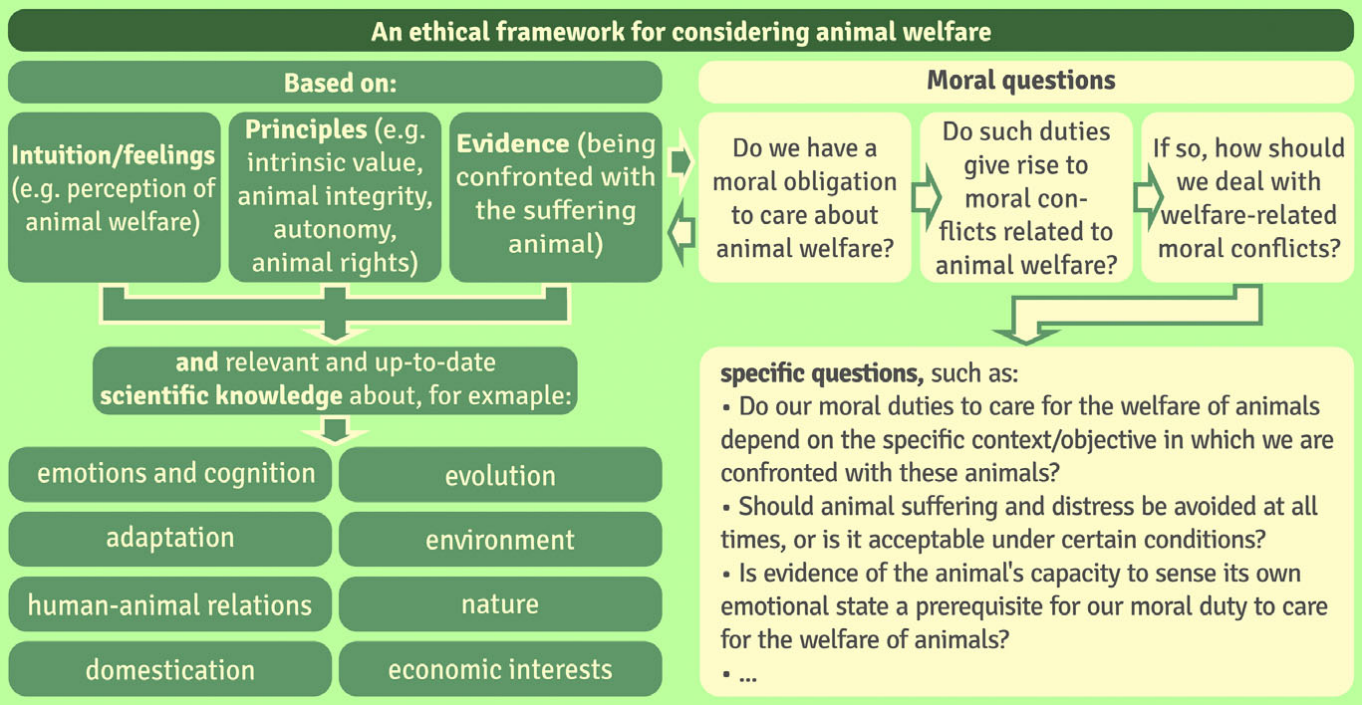
Framework for ethical considerations relating to the welfare of animals and the duty we owe to care for them (based on a modified figure from Ohl & van der Staay 2012 which, in turn, is based on a version originally published in Council on Animal Affairs 2012).

In order to provide solutions to perceived welfare problems, animal welfare scientists need to pay particular attention to two aspects: (1) the recommendations they make on animal welfare issues must be consistent with society’s moral values in order to generate sustainable approaches to animal welfare management; and (2) their considerations and recommendations must be based on the latest scientific knowledge. With this in mind, the Council on Animal Affairs (2012) in The Netherlands proposed an ethical framework to identify potential moral dilemmas in animal welfare and to inventory the factors influencing these dilemmas (see Figure 2). The framework aims to structure discussions on the ethical dimension of current and future animal welfare issues and outlines the steps needed to address and resolve these issues. It should be clear that the purpose of such a framework is to identify relevant ethical issues and potential moral dilemmas rather than to provide simple solutions. Note that this framework has been influenced by the definition of welfare by Ohl and van der Staay (2012; see also the updated version by Arndt *et al.* 2022).

Recently, Camenzind (2023) proposed a framework that can be used to determine the moral and normative position towards animals and their welfare, the 3D method. As the name suggests, this framework distinguishes three dimensions, namely *moral consideration*, *moral significance* and *moral practice*, which represent successive levels of ethical reasoning. The first level – moral consideration – is concerned with which species are regarded as morally relevant. The second level – moral significance – distinguishes between egalitarian and hierarchical variants of ethical frameworks, i.e. one species may be accorded a higher moral status than another (Kagan 2018). The third level – moral practice – deals with the content of moral obligations, such as how to implement respect for the moral status of an animal and how to establish concrete rules for moral action, such as how to treat animals appropriately (Schmidt 2011; Camenzind 2023).

### New ethics framework for the use of animals – beyond the context of the laboratory

#### From 3 to 12Rs

Regarding the ethical justification for using animals in research, the 4Rs extend the 3Rs by adding the principle of ‘Responsibility’ (Kiani *et al.* 2022) critically addressing the need for animal experimentation. The 12Rs are a further extension of the 3R and 4R principles, adding ethical constructs onto the design, conduct, analysis and reporting of animal experiments, thereby guiding stakeholders in the use of animals (Brink & Lewis 2023) (for a more detailed summary of the R principles, see Supplementary material).

#### Social benefit and animal welfare

According to DeGrazia and Beauchamp (2019), there is an urgent need for a guiding ethical framework for animal research. All stakeholders (see Figure 1) involved in animal research (including ethicists) are expected to be able to agree on this framework and to share the view that it is defensible; reasonable, i.e. realistic and acceptable; and practical. The framework consists of two core values, ‘social benefit’ and ‘animal welfare’, each of which contains three principles and is formulated from a human perspective. In relation to the core value of ‘societal benefit’, the principles should be met that: (1) there are no alternative non-animal methods to answer the scientific questions; (2) there is a net benefit to carrying out the research; and (3) the value of the research justifies the harm caused to the animals. With regard to the core value of ‘animal welfare’, the principles of: (1) no unnecessary harm; (2) meeting the animals’ basic needs; and (3) setting an upper limit to the harm that can be inflicted should be adopted.

#### Fundamental principles of biomedical animal research ethics

Tannenbaum (2017) summarised fundamental principles of animal research ethics, which address the question of how animal research should be conducted. They can also serve as a basis for formulating more concrete rules for the ethical conduct of animal research. In brief, with respect to animal experimental work, these principles state:

- Performing scientific research using animals is justified if its aim is to alleviate, and cure human disease, helps to alleviate pain, and suffering, distress, and prevents death;
- The acquisition and accumulation of knowledge necessary to improve the health and welfare of animals, including humans, requires *in vivo* experimentation on a wide range of animal species;
- Research should not cause more pain and distress to the animal than is necessary to achieve the scientific objectives of the study.

#### Farming of non-typical sentient animals

**Figure 3.**
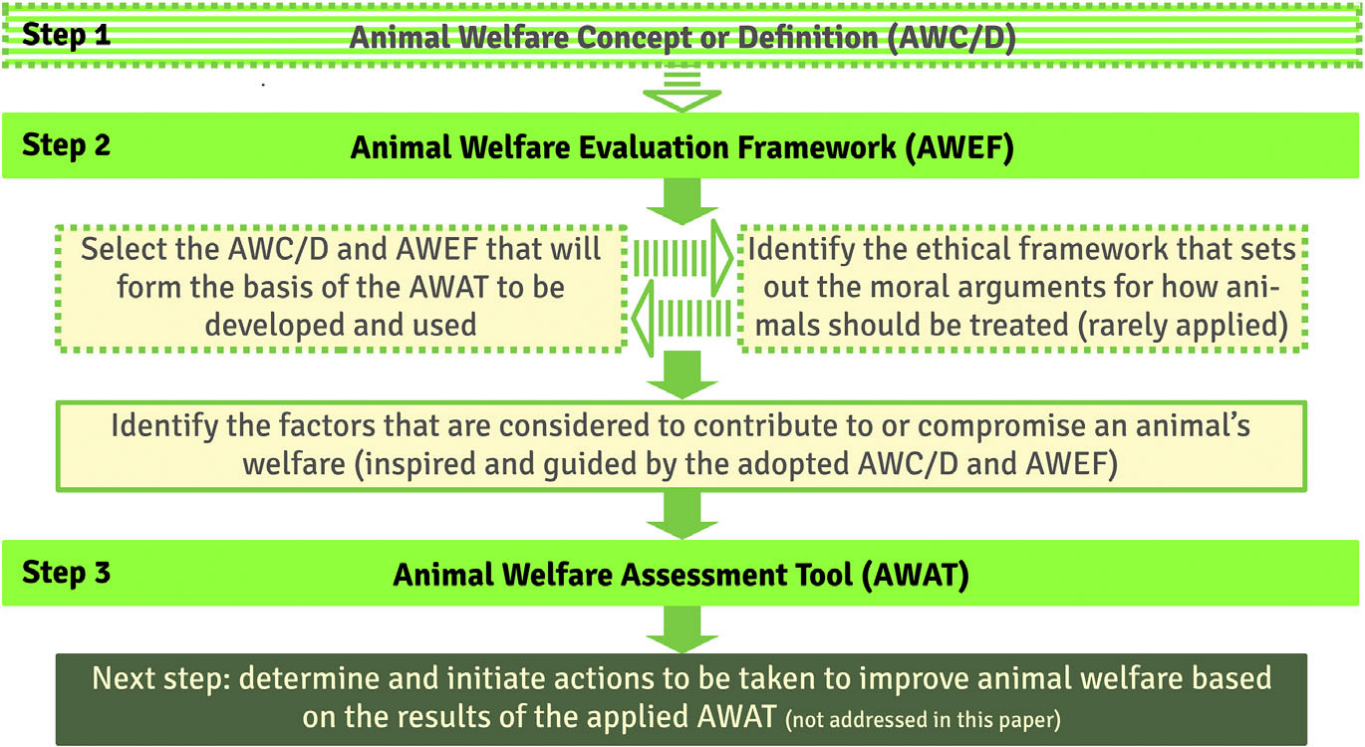
The development of tools for assessing animal welfare in three steps. The striped light green arrows and the dashed outlined area indicate processes that are rarely or never performed in practice or, alternatively, do not appear to have been explicitly reported in publications on the development of AWATs.

Recently, Mullan *et al.* (2024) presented a framework for addressing the question of whether the farming of non-typical sentient animals is ethical. The authors developed a decision tree that includes the key issues that need to be considered to answer this question. Their approach could be useful as an example or template for extending the question of how to deal with animal species that are less frequently kept by humans and for which there are gaps in our knowledge regarding their sentience. They argue, similarly to Arndt *et al.* (2024; Figure 3), that these gaps need to be filled by scientific research, as sentience is a crucial aspect to consider in ethical evaluations of animal welfare and animal integrity (Arndt *et al.* 2024).

### Towards a broader framework for the ethical use of animals?

The above-described frameworks represent examples that take into account differences in societal values and ethics. However, the developers of the described ethical frameworks refer to different sets of key characteristics and moral values, i.e. they take different positions in animal ethics and give different weight to different components, although they obviously build on and integrate concepts from well-established frameworks such as the 3Rs, the Five Freedoms and the Five Domains model. Their positions on animal ethics are not always explicitly stated, and it may be useful to identify the underlying ethical positions reflected by the different ethical frameworks, for example, by using Camenzind’s 3D method (Camenzind 2023) (see previously).

Perhaps components of these frameworks could be combined, adapted and extended to provide an ethical framework for all animals, with a distinction eventually being made between sentient and non-sentient species, since animal welfare requires that animals be sentient (see also Arndt *et al.* 2024). We suggest that such a framework should be part of the considerations for use in all contexts where animals are kept for human purposes, whether for research, consumption or companionship.

## Animal welfare assessment tools (AWATs)

Ideally, once an explicit agreement on the definition/concept and framework for animal welfare has been reached in a first step and communicated transparently and clearly between stakeholders, the next step is to develop assessment tools. In practice, however, the first step is usually skipped or not considered in publications on the development of an AWAT. Instead, two other approaches are commonly used to derive AWATs.

The first approach refers more or less explicitly to existing AWEFs from which AWATs or protocols are derived and validated to measure the welfare of animals (as individuals or in groups) (e.g. see van Eerdenburg *et al.* 2021; Table 1 for a list of tools used to measure cow welfare). A further example is the Animal Welfare Assessment Grid, a visual mapping tool for assessing animal welfare, originally developed for zoo animals (Jones *et al.* 2022) which has been extended to a wide range of species kept in different environments (e.g. companion animals, Dunn 2020; laboratory animals, Honess & Wolfensohn 2010; Malkani *et al.* 2022; invertebrates such as decapodes and encephalopods, Narshi *et al.* 2022).

The second approach is based on expert panel surveys, which capture the facets that experts believe contribute to the welfare of specific species kept under specific husbandry and management conditions (e.g. animals kept in zoos, Jones *et al.* 2022; free ranging animals, such as dolphins, Serres *et al.* 2024), i.e. usually covering a combination of animal-, resource-, and management-based parameters (Czycholl *et al.* 2015). Data collected in a survey or from existing surveys are subjected to a statistical technique, such as factor analysis, to decide which items should be included in the AWAT. This approach requires the collection of a considerable amount of data on locations (farm, zoo, etc) which are then integrated into a welfare score that is believed to be a reliable reflection of an animal’s welfare state (Wemelsfelder & Lawrence 2001).

Another commonly used method is the ‘Delphi consultation’ approach, consisting of a multi-round process of questionnaires and controlled feedback on items related to animal welfare. A heterogeneous panel of experts, presumably representing stakeholders from different backgrounds, participate anonymously; the aim of this process being to reach a group consensus on animal welfare. The stability of responses between rounds is a proxy for the consensus reached (Truelove *et al.* 2020). The Delphi consultation approach has been used to identify welfare indicators for a wide range of animal species (from reptiles to primates, e.g. Campos-Luna *et al.* 2019; Rioja-Lang *et al.* 2020; Truelove *et al.* 2020; Whittaker *et al.* 2021; Piseddu *et al.* 2024).

Animal welfare assessment tools include several domains/categories that encompass both environmental aspects, as the environment provides hazards and opportunities for animals to reach a positive welfare state, and animal-based indicators that reflect the current welfare state of the individual. The best known AWATs are the Welfare Quality® welfare assessment protocols (developed in a large EU-funded project; Blokhuis *et al.* 2010), of which specific versions have been developed to measure the welfare of different farm animal species (pigs: Dalmau *et al.* 2009; cattle: Kirchner *et al.* 2014; poultry: Welfare Quality®, assessment protocol for poultry [broilers, laying hens] 2009). The ‘Animal Welfare Indicators (AWIN)’ project (7th Framework programme 2024) was another major research project funded by the EU, that, similar to the Welfare Quality® protocols, aimed to improve animal welfare by developing welfare assessment protocols that could be used on-farm. Depending on the target species, the protocols have been adapted to include species-specific elements; they have, for example, also been adapted to assess the welfare of non-agricultural animals (e.g. reptiles; Benn *et al.* 2019) and animals living under different housing and management conditions in different climatic regions (e.g. cattle, Hernandez *et al.* 2017; Kaurivi *et al.* 2019, or sheep raised extensively in the southern hemisphere, Munoz *et al.* 2019; Willis *et al.* 2021).

Wemelsfelder (2007) took a different approach to assessing animal welfare with the development of the ‘Qualitative Behavioural Assessment’ method. The method has been developed within the context of ‘Quality of Life’ and is based on the free-choice profiling procedure (for details, see Wemelsfelder & Lawrence 2001). Whereas most AWATs use a fixed list of items that are thought to reflect (aspects of) an animal’s welfare, observers using the Qualitative Behavioural Assessment are asked to generate a list of welfare descriptors themselves, such as: calm, anxious, timid or confident (Wemelsfelder & Lawrence 2001), all based on behavioural expressions. The authors argue that the expression of these descriptors over time reflects changes in conditions with greater sensitivity than a list of fixed items in conventional assessment tools (Wemelsfelder *et al.* 2000).

## Distinction between animal welfare definitions and concepts, animal welfare evaluation frameworks, and animal welfare assessment tools

Animal Welfare Definitions and Concepts articulate a theoretical concept of animal welfare (at the intersection of ethics and science; see e.g. Arndt *et al.* 2024) ranging from a single factor to a multifactorial view. AWEFs identify concrete aspects that should allow for a qualitative and quantitative assessment of animal welfare and initiate the search for practical solutions that can be implemented and whose impacts can be measured with validated measurement tools. Finally, AWATs consist of lists of items that allow for the qualitative or quantitative assessment of different aspects that are considered to be relevant to the welfare of an animal (see Figure 3).

Items dealing with different aspects of the same topic (e.g. housing conditions, hygiene, health) are listed under a ‘chapter’, which summarises the items of that topic. The total score per chapter and/or the sum of all chapters will normally be taken as the resulting welfare score. However, it remains to be demonstrated that they actually reflect ‘welfare’. An animal welfare assessment tool is a set of questionnaires and other measurement tools designed to quantify the welfare of an individual or group. What is understood as ‘welfare’ is therefore determined by the items that make up the welfare assessment tool, and how the various items are weighted. Without a proper definition of welfare and without a common understanding of what welfare means, it is not too surprising that the results of different welfare assessment tools may show poor correlations.

## What about animal species that do not fall under animal welfare definitions?

Current definitions of animal welfare are limited to sentient species. However, as Nussbaum notes, ”*life forms don’t line up to be graded on a single scale: they are just wonderfully different*“ (Nussbaum 2018; p 5). Furthermore, ”*we need theoretical approaches that are sound in terms of reality, grappling with what we know about animals, and that also direct law in a useful fashion*” (Nussbaum 2018; p 3). We might add that they must underpin the formulation of guidelines for action towards all species, sentient and non-sentient. Recently, following the ideas of others (e.g. Rutgers & Heeger 1999), we have proposed that the principle of animal integrity be applied to all animal species, including those that lack the capacity to experience negative and positive emotions (Arndt *et al.* 2024). This proposal is based on a zoocentric or biocentric perspective, which provides its theoretical and ethical framework. We have defined animal integrity as follows: *“An animal’s integrity is most likely to be intact when its wholeness, its species-specific balance and its ability to sustain itself independently in an environment appropriate to its species are ensured, i.e. when its environment provides the resources necessary for survival and reproduction, and when the animal has the ability and opportunity to cope appropriately with the challenges of the [prevailing] environmental circumstances”* (Arndt *et al.* 2024; p 7).

## Discussion

The development and validation of animal welfare assessment tools can be seen as a three-step process, starting with the formulation of AWC/Ds, followed by the formulation of an appropriate AWEF.

### Do we need animal welfare concepts and definitions?

When discussing animal welfare, it is advisable to provide a definition of what is meant by the term, as there is still no generally accepted understanding of this concept (Botreau *et al.* 2007; Carenzi & Verga 2009; Fisher 2009). The definition adopted should capture the most relevant components by which animal welfare can be described and contribute to the formulation of criteria against which animal welfare can be measured. This set of criteria should have a number of characteristics: It should be exhaustive, i.e. it should cover all relevant aspects of animal welfare, it should be limited to the most important elements, it should ideally be accepted by all stakeholders and the number of its elements should be limited (Botreau *et al.* 2007). We are aware that this is going to be difficult to achieve.

In contrast, Fisher (2009) argues that a variety of definitions, reflecting different scientific positions and ethical viewpoints, may be important in considering animal welfare, some of which may be overlooked and ignored if only one definition is adhered to. Rault and co-authors (2020) deplore the practice of the vast majority of papers being ambiguous or silent about their position or definition. As a result, it often remains unclear which underlying concept of animal welfare was implemented, whereas definitions of animal welfare can help to ensure a common understanding (Bacon *et al.* 2021). As Camerlink notes, “*The need for a unified definition becomes particularly important when it influences international law and regulations*” (Camerlink 2022; pp 1–2). Unfortunately, to our knowledge, the influence of animal welfare definitions on the drafting of regulations and legislation appears to be very limited, or is not explicitly referred to in practice.

The AWC/Ds can be used to formulate an AWEF and deduce specific hypotheses about the factors (not a list of items) that contribute to animal welfare. The contribution of the proposed relevant elements to animal welfare, either individually or in combination, should be investigated in scientific studies (see, e.g. Browning 2022) and validated, which does not seem to be common practice (e.g. Welfare Quality®, see European Commission 2009; p 3). Moreover, the distinction between AWC/Ds and AWEFs is rarely considered in animal welfare science; rather, it seems to be common practice that many, if not all, AWEFs have been developed without reference to a particular AWC/D and are derived from, or loosely based on, (mostly undefined) notions of what animal welfare is (e.g. Mellor & Reid 1994; Botreau *et al.* 2007). Admittedly, the absence of a reference to an AWC/D does not exclude the possibility that a welfare definition has been implicitly adopted. Nevertheless, AWEFs sometimes seem to be seen as a substitute for proper definitions when developing AWATs: welfare is defined by the various aspects/chapters/items measured by the AWATs.

### The significance of ethics frameworks

Our treatment of animals is, implicitly or explicitly, guided by our own moral position. Ethical frameworks help us to define and clarify our moral position. While definitions of animal welfare influence the way we perceive the factors that are thought to affect animal welfare, awareness of the ethical position guides our thinking about animal welfare and influences our decisions about how to interact with and treat animals. As Rollin argued, “[…] *which ethical framework one adopts will in fact determine the shape of science studying animal welfare*” (2015; p 761).

Although several ethical frameworks have been developed (see previously), they are often neglected in the animal welfare literature. Summer (1986) offers a possible explanation for this: Philosophers are the experts on moral issues, a topic of great importance to animal welfare scientists, among others. However, the non-philosophical audience may find it difficult to follow the philosophers’ reasoning and may therefore pay less attention to the ethical reasoning than is desirable. On the other hand, when philosophers write for a wider audience, their texts may become lengthy (too lengthy for the non-philosopher interested in the topic, and too layman-like for philosophers). The result being that even a more accessible philosophical/ethical text will find a limited audience of philosophers and non-philosophers alike. This is reflected in the relative lack of discussion of ethical issues in the animal welfare literature.

The 3D method of Camenzind (2023) serves exclusively as a framework for analysing and determining the normative position of scientists on animal welfare whereas, for example, the framework proposed by the Council on Animal Affairs (2012) and Fraser and McRae’s ‘Harms model’ (2011) includes both ethical and scientific issues. One of the reasons that the results of different AWATs may diverge rather than be highly correlated may be the heterogeneity of the implicit and explicit ethical concepts behind many AWEFs and AWATs (see e.g. van Eerdenburg *et al.* 2021). As a result, different AWATs may reflect different welfare concepts, with a wide variation in the extent to which they overlap in the components considered to be relevant and characteristic of welfare.

### The purpose of animal welfare evaluation frameworks

AWEFs provide a structure for evaluating animal welfare (Bacon *et al.* 2021). An AWEF can specify the aspects which are thought to crucially represent the elements of animal welfare on the conceptual basis of an AWC/D (see e.g. Table 1 in Jones *et al.* 2022). Following Imenda’s (2014) categorisation, most, if not all, current AWEFs can be understood as conceptual frameworks, i.e. they do not appear to be based on a specific animal welfare concept or definition.

Based on an AWEF, an animal welfare measurement tool (consisting of observables and measureables; van der Staay *et al.* 2009) can be developed and validated. In a discussion of AWEFs such as the Five Freedoms and the Five Domains, Webster concludes that “*as far as animals are concerned, it is not what we think that counts but what we do, I suggest that they are likely to have a more general impact*” (Webster 2016; p 1).

In addition to the Five Freedoms, the Five Domains model and the Quality of Life concept, there are other candidate concepts that could serve as AWEFs. The adoption of new AWEFs could open up new perspectives on animal welfare and its measurement. An innovative perspective can lead to new insights. For example, it may be worth considering the utility of Nussbaum’s Capability approach by (Robeyns 2005; Allmark & Machaczek 2015) and of the Exposome Concept (Kalia *et al.* 2020; Vermeulen *et al.* 2020; Arndt *et al.* 2022) as AWAFs. While the exposome concept might focus attention on environmental influences, the capabilities approach might focus attention on other welfare-relevant aspects that otherwise escape attention.

Supplementing animal welfare frameworks with information on the underlying ethical and moral considerations, e.g. through the use of an ethics framework, can add depth. It is conceivable that the inclusion of ethical considerations may draw attention to issues that would otherwise be inadequately addressed or ignored (see also Brown 2014).

### The purpose of animal welfare assessment tools

Due to the apparent diversity of AWEFs behind the development of some of the most commonly used AWATs, and the fact that AWATs are not based on a consensual AWC/D (i.e. one preferably agreed upon by all stakeholders, including consumers and citizens concerned about animal welfare), AWATs may measure different things. AWATs based on stakeholder expert opinion may suffer from other but equally important shortcomings: The experts and stakeholders may have (completely) different perspectives on ‘animal welfare’ and represent very different interests; the selection of experts and the assessment of the relevance of their expertise may be biased and lack transparency (see also Browning 2022); experts and stakeholders may act as lobbyists for particular interested third parties; those who shout the loudest and have the best lobby may have the greatest influence on AWATs.

From a scientific point of view, there are two additional critical points: they concern issues of testability and reproducibility (Hampton 2023). This makes it difficult to compare the results of animal welfare scores derived from different AWATs, and may be two of the reasons why correlations between welfare scores derived from different AWATs are sometimes reported to be low or absent (e.g. Andreasen *et al.* 2013; van Eerdenburg *et al.* 2021).

A clear reference to a particular AWC/D will guide the choice of a particular AWAT and, based on the results of the welfare assessment, the type of action to be undertaken to intervene on behalf of the animals, i.e. if monitoring reveals compromised or impaired welfare, the causes must be investigated and action taken to address them. Many AWATs already exist, and their number is increasing. AWATs based on other than the commonly used AWEFs could greatly enrich the list of aspects for the assessment of animal welfare and stimulate the expansion of the repertoire of assessment methods for determining the state of animal welfare. It should be noted that even when people agree that they have a responsibility for animal welfare and the results of an assessment tool indicate that an animal’s welfare is at risk, their willingness to intervene on the animal’s behalf can vary widely. Animal welfare assessment therefore draws our attention to the welfare status of individual animals or groups of animals, but does not guarantee that appropriate action will be taken, because “*In practice, interpretation of welfare status and its translation into the active management of perceived welfare issues are both strongly influenced by context and, especially, by cultural and societal values*” (Ohl & van der Staay 2012; p 13). Webster (2016) pointed out that “*the most important purpose of any welfare monitoring system is to identify and address specific problems*” (2016; p 4). Interventions that address identified problems (for examples, see Nordquist *et al.* 2017) can then be evaluated, and the impact of interventions to address these problems can be measured.

## Animal welfare implications and conclusion

The development of AWATs could benefit from an explicitly defined conceptual or theoretical background at the outset, including disclosure of the underlying ethical position. This approach will help to provide a more generally accepted direction for the discussion of animal welfare as well as increasing the probability of consensus about animal welfare research and the development of AWATs. In this process, more clarity is needed regarding the roles of all stakeholders with regard to their input and responsibilities in discussing animal welfare issues and formulating animal welfare goals and guidelines.

It is expected that a common definition of animal welfare, once endorsed by the majority of all stakeholders, including consumers, but especially experts and animal welfare scientists, will facilitate agreement on (most) aspects considered relevant to animal welfare. It will facilitate the clarification of the underlying concepts and their ethical basis, as well as the identification of research topics and priorities for issues to be addressed in animal welfare research.

It will also facilitate the formulation of guidelines and policies for the proper treatment of animals and speed up the translation of scientific knowledge and it implementation into practice. In particular, we outline the opportunities for the framework to be applied across contexts in which animals are kept, as the basic welfare needs of an individual will be the same, notwithstanding that the environment (e.g. farm, zoo, climatic zone) and its inherent stressors may differ. Therefore, different disciplines such as farm and laboratory animal science can benefit from developments in each of these areas.

## Supporting information

van der Staay et al. supplementary material van der Staay et al. supplementary material

## Supplementary material

The supplementary material for this article can be found at http://doi.org/10.1017/awf.2025.23.
