# Supplementary material for: Animal welfare definitions, frameworks, and assessment tools: Advancing the measurement and laying the foundation for improved animal welfare through a three-step approach
Source: Anim Welf. 2025 May 7;34:e30. doi: 10.1017/awf.2025.23 (PMC12075010; doi:10.1017/awf.2025.23)
Supplement: van der Staay et al. supplementary material [file S0962728625000235sup001.pdf]

Supplementary material

Animal welfare definitions, frameworks, and assessment tools

— Advancing the measurement and laying the foundation for improved animal welfare through a three-step approach

F. Josef van der Staay[1,3], Vivian C. Goerlich[2], Franck L. B. Meijboom[4] & Saskia S. Arndt[2]*

FJS: https://orcid.org/0000-0002-8704-3366

VCG: https://orcid.org/0000-0002-8586-4435

FLBM: https://orcid.org/0000-0002-0752-016X

SSA: https://orcid.org/0000-0002-3678-1158

[1] Department of Population Health Sciences, Division of Farm Animal Health, Behaviour and Welfare Group (Formerly: Emotion and Cognition Group), Faculty of Veterinary Medicine, University Utrecht, Utrecht, The Netherlands

[2] Department of Population Health Sciences, Division of Animals in Science and Society, Animal Behaviour Group, Faculty of Veterinary Medicine, Utrecht University, Utrecht, The Netherlands

[3] University Medical Center (UMC) Utrecht, Brain Centre, Utrecht, The Netherlands

[4] Department Population Health Sciences, Division of Animals in Science & Society, Sustainable Animal Stewardship, Faculty of Veterinary Medicine, University Utrecht, Utrecht, The Netherlands

* Corresponding author:

Saskia S. Arndt, PhD, Division of Animals in Science and Society, Animal Behaviour Group, Faculty of Veterinary Medicine, Utrecht University, PO Box 80166, 3508 TD Utrecht, The Netherlands,

Tel : +31 (0) 639585472, Email : s.s.arndt@uu.nl

This supplementary text summarises the development of the 3R's in recent decades. Originally conceived as a set of principles to which animal research should adhere to ensure scientific quality, the 4 and 12 Rs also include ethical and societal considerations. Although defined for laboratory animals, these principles can be applied in any context where animals are used for human purposes, e.g. as a source of food. (Rault *et al.* 2022).

Three R's and more

The 3Rs

The aim of the 3Rs is to avoid or reduce pain and distress in laboratory animals. Using fewer animals (reduction), avoiding or eliminating distress (refinement), and using unconscious rather than conscious animals (replacement) constitute the three principles of the 3Rs (Russell and Burch 1959; Tannenbaum 2017; Tannenbaum and Bennett 2015). The 3Rs do not address the ethical justification of animal research (Louis-Maerten *et al.* 2024) and do not advocate the cessation of animal experimentation, provided the aim of the study is to advance science and medicine (Tannenbaum and Bennett 2015). Since the publication of the 3Rs and building on their principles, new and broader ethical frameworks for the use of animals in research have been proposed. These have been driven by the recognition that the 3Rs do not adequately address some fundamental ethical issues, including the moral justification and responsibility for animal experimentation (DeGrazia and Beauchamp 2019).

The 4Rs

By adding a fourth principle, 'responsibility', the 4Rs address the ethical justification for using animals in research (Kiani *et al.* 2022), i.e. whether animal experimentation is necessary for the advancement of biomedicine. Responsibility advocates "integrity, honesty and scientific correctness in the appropriate and reasonable use of laboratory animals" (Arora *et al.* 2011, p. 2).

The 12Rs

The 12Rs further add several research ethics constructs, integrating these into a unified, comprehensive framework to guide stakeholders in animal use (Brink and Lewis 2023). In short the 12Rs consist of i) Animal Welfare Rs (AWRs), 2) Social Value Rs (SVRs), 3) Scientific Integrity Rs (SIRs), and 4) Domain intersecting Rs (DIR's) (Fig. 2 in Brink and Lewis 2023 shows an overview graphic of this framework).

- The **AWRs** are presented by the 3Rs (**R**eduction, **R**efinement, and **R**eplacement) (Russell and Burch 1959).
- The SVRs reflected the use of sound research methods and techniques, a prerequisite for obtaining reliable and valid data that are relevant to answering research questions. In terms of content, these are largely covered by the principles of the Five Domains and Five Freedoms and are labelled **R**espect, **R**esponsibility and **R**egulation.
- The **SIRs** contain the principles **R**eproducibility, **R**elevance, and T**R**ansferability. "Reproducibility" refers to the use of appropriate Material and Methods to conduct the study, to analyse and generalise the results, and to share all relevant information in such a way that the study can be replicated. "Relevance" of the study refers to the extent to which animals, humans and society benefit from the results of a study. Transferability" or "translatability" may refer to the generalisability of the study and, if animal models are used, the degree to which face, predictive, construct and other validity criteria are met.
- Finally, a fourth set of R's are the **DIRs** with the principles **R**ighteousness, **R**eliability, and **R**eckoning. "Righteousness" at the intersection of SIR and AWR is the pursuit of respectable science in which scientists are fair, good, noble, dignified and serious; "Reliability" at the intersection of SIR and AWR refers to the robustness, quality, trustworthiness and applicability across settings or contexts, and generalisability of the results obtained and conclusions drawn, embedded in a culture of scientific quality and integrity; "Reckoning", at the

intersection of SIR and AWR, refers to accountability through actions taken during the design and conduct of an animal study and after its completion.

Brink and Lewis (2023) advise that the 12Rs framework should be applied throughout the process of designing, conducting, analysing and reporting animal studies. Because it takes into account differences in societal values and ethics, it can provide a template or model for a universally applicable ethical framework for all who work with animals. Therefore, it is conceivable to consider whether this framework could be adapted and extended to cover all areas where animals are kept under human responsibility.

References

Arora T, Mehta AK, Joshi V, Mehta KD, Rathor N, Mediratta PK and Sharma KK (2011) Substitute of animals in drug research: an approach towards fulfillment of 4R's. *Indian Journal of Pharmaceutical Sciences* **73**(1), 1–6. https://doi.org/10.4103/0250-474X.89750.

Brink CB and Lewis DI (2023) The 12 Rs framework as a comprehensive, unifying construct for principles guiding animal research ethics. *Animals* **13, 1128**, 14. https://doi.org/10.3390/ani13071128.

DeGrazia D and Beauchamp TL (2019) Beyond the 3 Rs to a more comprehensive framework of principles for animal research ethics. *ILAR Journal* **60**(3), 308–317. https://doi.org/10.1093/ilar/ilz011.

Kiani AK, Pheby D, Henehan G, Brown R, Sieving P, Sykora P, Marks R, Falsini B, Capodicasa N, Miertus S, Lorusso L, Dondossola D, Tartaglia GM, Ergoren MC, Dundar M, Michelini S, Malacarne D, Bonetti G, Dautaj A, Donato K, Medori MC, Beccari T, Samaja M, Connelly ST, Martin D, Morresi A, Bacu A, Herbst KL, Kapustin M, Stuppia L, Lumer L, Farronato G and Bertelli M (2022) Ethical considerations regarding animal experimentation. *Journal of Preventive Medicine and Hygiene* **17;63**(2 Suppl 3), E255–E266. https://doi.org/10.15167/2421-4248/jpmh2022.63.2S3.2768.

Louis-Maerten E, Rodriguez Perez C, Cajiga RM, Persson K and Elger BS (2024) Conceptual foundations for a clarified meaning of the 3Rs principles in animal experimentation. *Animal Welfare* **33, e37**, 1–11. https://doi.org/10.1017/awf.2024.39.

Rault J-L, Binder R and Grimm H (2022) Rethink farm animal production: the 3Rs. *Science* **378, 6622**, 842. https://doi.org/10.1126/science.adf3351.

Russell WMS and Burch RL (1959) *The principles of humane experimental technique*. London: Methuen; Reprinted by UFAW, 1992: 8 Hamilton Close, South Mimms, Potters Bar, Herts EN6 3QD England. ISBN 0 900767 78 2. http://altweb.jhsph.edu/pubs/books/humane_exp/het-toc

Tannenbaum J (2017) Ethics in biomedical animal research: the key role of the investigator (chapter 1). In Conn PM (ed), *Animal models for the study of human disease*, 2nd edn. London, United Kingdom: Elsevier Academic Press, 3–46.

Tannenbaum J and Bennett BT (2015) Russell and Burch's 3Rs then and now: the need for clarity in definition and purpose. *Journal of the American Association for Laboratory Animal Science* **54**(2), 120–132.
